# Calcium-Mediated Control of Polydopamine Film Oxidation and Iron Chelation

**DOI:** 10.3390/ijms18010014

**Published:** 2016-12-22

**Authors:** Luke Klosterman, Christopher J. Bettinger

**Affiliations:** 1 Department of Materials Science and Engineering, Carnegie Mellon University, Pittsburgh, PA 15213, USA; lkloster@andrew.cmu.edu; 2 Department of Biomedical Engineering, Carnegie Mellon University, Pittsburgh, PA 15213, USA

**Keywords:** polydopamine, melanin, thin film, metal cation, dopamine oxidation

## Abstract

The facile preparation of conformal polydopamine (PDA) films on broad classes of materials has prompted extensive research into a wide variety of potential applications for PDA. The constituent molecular species in PDA exhibit diverse chemical moieties, and therefore highly variable properties of PDA-based devices may evolve with post-processing conditions. Here we report the use of redox-inactive cations for oxidative post-processing of deposited PDA films. PDA films incubated in alkaline CaCl_2_ solutions exhibit accelerated oxidative evolution in a dose-dependent manner. PDA films incubated in CaCl_2_ solutions exhibit 53% of the oxidative charge transfer compared to pristine PDA films. Carboxylic acid groups generated from the oxidation process lower the isoelectric point of PDA films from pH = 4.0 ± 0.2 to pH = 3.1 ± 0.3. PDA films exposed to CaCl_2_ solutions during post-processing also enhance Fe^2+^/Fe^3+^ chelation compared to pristine PDA films. These data illustrate that the molecular heterogeneity and non-equilibrium character of as-deposited PDA films afford control over the final composition by choosing post-processing conditions, but also demands forethought into how the performance of PDA-incorporated devices may change over time in salt solutions.

## 1. Introduction

The autoxidation of dopamine in alkaline aqueous solutions produces a dark insoluble material known as polydopamine (PDA). PDA synthesis produces particles in solution and conformal nanometer scale films on various substrate materials [1]. Catechol-bearing PDA exhibits robust surface adhesion [2,3], metal chelation [4], and electrooxidative control over redox states [5]. These unique properties have prompted PDA’s exploration for numerous applications [6,7] in antifouling surfaces [8,9], biointerfaces [10,11], and high surface-area aqueous metal sorption devices [12,13,14].

The primary means of controlling the intrinsic properties of PDA films thus far explored have been the conditions of the precursor solution. The intrinsic oxidation mechanism of dopamine is sensitive to dissolved O_2_, dopamine, and hydronium concentration [15], however buffer composition and substrate chemistry also influence PDA film growth [16,17]. The rate of PDA film growth is accelerated as pH values approach 9.5 above which (pH > 9.5) increased PDA solubility compromises film quality [17,18]. Copper ions and periodate oxidants, in contrast to dissolved O_2_, can create PDA films in aqueous solutions at pH < 7.0 [16,19]. Electrochemical oxidation of dopamine produces PDA films with increased ion permeability [20] and stability [21] compared to PDA films formed through autoxidation. Buffer composition and substrate chemistry also influence the deposition rate and nanostructure of PDA films [17,22,23]. Post-synthesis treatments such as thermal annealing [11,24] or FeCl_3_ treatments [25] of PDA films increase their stability in alkaline solutions compared to pristine PDA films. Taken together, these numerous strategies illustrate the use of PDA deposition conditions and post-processing to tune PDA composition and reactivity.

A comprehensive understanding of structure-processing-property relationships in PDA could accelerate the implementation of this material in many technologies. Devices incorporating PDA, such as bio-interface coatings [10,11] and ion-exchange coatings [12,14], are most commonly applied in aqueous solutions of metal salts (e.g., body fluid and environmental waters). However, there is little knowledge regarding the temporal evolution of PDA properties in response to solutions of group I and II cations which are known to influence cohesive forces [26] and redox kinetics [27] in other catecholic materials. Herein, we report the influence of CaCl_2_ solutions on oxidation of pre-deposited PDA films. The oxidation kinetics, redox state, and physicochemical properties of PDA films are reported. Functional properties of PDA films including catechol-cation chelation are also investigated.

## 2. Results and Discussion

### 2.1. Alkaline Calcium Solutions Promote Generation of Semiquinone Radicals and Oxidative Evolution of Polydopamine (PDA) Films

**Scheme 1:**
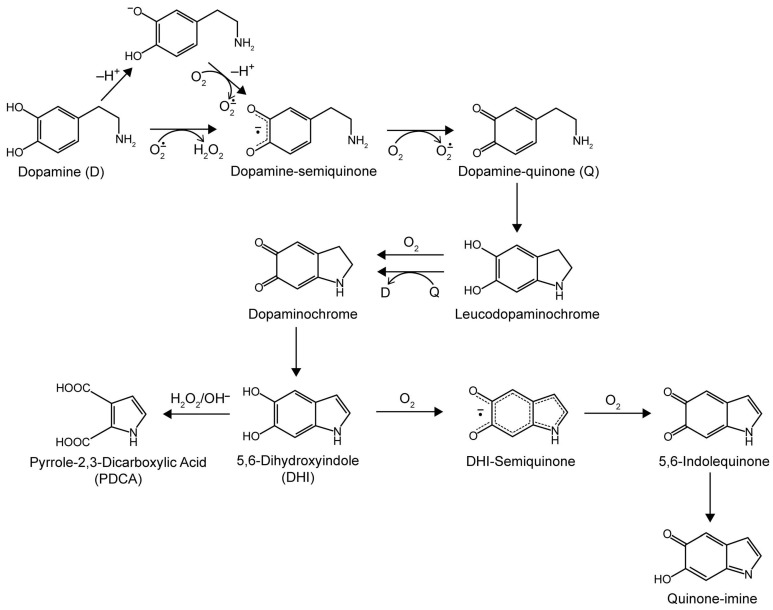
Oxidation pathway of dopamine in alkaline solutions exposed to O_2_. The proposed structure of polydopamine consists of oligomers of all species shown [22,28,29].

Oxidative chemical synthesis of PDA films from dopamine is a complex multi-step process (Scheme 1) that includes redox processes, cyclization, polymerization, and various cleavage reactions that may serve as control points for altering the composition of PDA [15,22,28]. Although the intermediates are relatively simple, multiple different possible reaction pathways quickly increase the diversity of products. The rate limiting step is the first 1e^–^ oxidation of deprotonated catechol by molecular O_2_ to produce dopamine-semiquinone and O_2_^–^ [15]. The semiquinone is oxidized again by O_2_ to produce dopamine-quinone, which can then undergo intramolecular cyclization. Subsequent oxidation steps and intramolecular rearrangements produce various heterocyclic species [22,28,29]. H_2_O_2_ generated upon oxidation of the catechol groups by O_2_^–^ can also generate pyrrole-carboxylic acids by cleaving 5,6-dihydroxyindole units [22,30]. Reactive monomers produced along the dopamine oxidation pathway oligomerize (observed up to the octamer level in PDA [22,28] or 30-mer for solely 5,6-dihydroxyindole (DHI) polymerization [31]) and aggregate into disordered graphite-like nanostructures [32] akin to naturally occurring melanins [33,34]. The resulting PDA has a stable population of free radicals (ca. 1 radical per 1700 monomers [35]) that depends on the buffer used during PDA synthesis [23].

**Figure 1:**
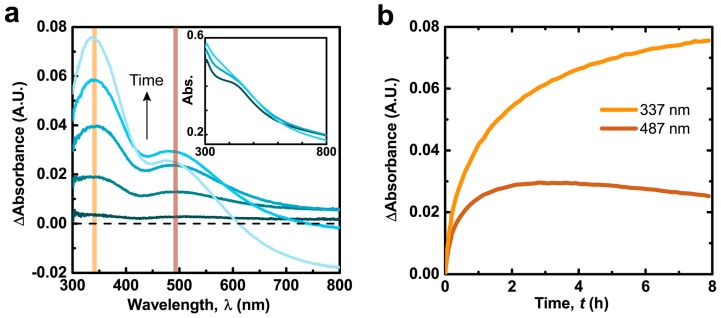
(**a**) Subtracted ultraviolet-visible (UV-Vis) spectra of polydopamine (PDA) film in 300 mM CaCl_2_, 50 mM tris(hydroxymethyl) aminomethane (Tris) buffer pH = 9.5. Inset: Original spectra; (**b**) Absorbance at the designated wavelengths over time (spectra recorded every 83 s).

Freshly-synthesized PDA films and particles are partially oxidized and contain species from all stages of the dopamine oxidation pathway [22,28]. Therefore, the chemical composition (and subsequent physical properties) of PDA films may evolve continuously over their lifetime as constituent molecules are exposed to controlled conditions after film deposition or de facto post-processing conditions via the ambient environment. The in situ chemical evolution of PDA films incubated in CaCl_2_ solutions was monitored via ultraviolet-visible (UV-Vis) spectroscopy. The difference in the spectra of the PDA films from the initial recording (Δ*A* = Abs(*t*) − Abs(0 min)) reveal two peaks at *λ*_Abs_ = 337 nm and *λ*_Abs_ = 487 nm (henceforth designated as Δ*A*_337nm_ and Δ*A*_487nm_) and a tail extending through the visible region (Figure 1a). The Δ*A*_337nm_ peak is assigned to ortho-semiquinone radicals (typically observed in the *λ*_Abs_ = 300–350 nm region [36,37,38,39]). The Δ*A*_487nm_ peak may represent convoluted contributions from dopaminochrome (*λ*_Abs_ = 476 nm) [40] and the delocalized radical of DHI-semiquinone (*λ*_Abs_ = 490 nm) [37], both of which are oxidation products of dopamine. The absorbance tail through the visible region is attributed to intermolecular perturbations of convoluted oligomerization products (used to explain the black color of eumelanins) [41,42,43].

The hour long time scales for the spectral development of CaCl_2_-incubated PDA films (Figure 1b) suggests an oxygen kinetic limitation, considering that the autoxidation of dopamine solutions occurs on similar time scales (Figure S1) [15,18]. Over the course of several hours the two peak intensities increase and reach a maximum (*t*_max_ = ~11 and 3 h for Δ*A*_337nm_ and Δ*A*_487nm_ respectively, Figure S2) then decrease. The loss of absorption and differences in temporal development of the two peaks is attributed to peroxidative degradation of species with PDA (referred to as bleaching) [44], which has been observed to more strongly affect absorption in the red region for synthetic melanin [45]. Additionally, the simple 1e^−^ oxidative generation of semiquinones from the numerous catechol species in PDA could lead to their more rapid and extensive generation (and increase in Δ*A*_337nm_) compared to more advanced oxidation products (e.g., dopaminochrome, DHI-semiquinone) exhibiting visible chromophores (observed via Δ*A*_487nm_).

**Figure 2:**
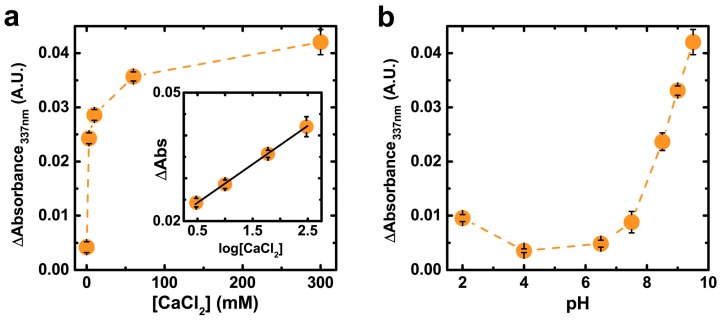
(**a**) Subtracted spectra absorbance at *λ*_Abs_ = 337 nm after 1 h as a function of [CaCl_2_] in 50 mM Tris buffer pH = 9.5; (**b**) Subtracted spectra absorbance after 1 h as a function of pH in 300 mM CaCl_2_, 50 mM Tris buffer. See Figure S5 for equivalent data at *λ*_Abs_ = 487 nm. Data are represented as the average ± SD (*n* = 2).

The temporal evolution of PDA film composition is likely due to altered oxidative mechanisms induced by Ca^2+^ cations. Previous investigations have demonstrated that Ca^2+^ ions accelerate the oxygen consumption of solutions of pyrocatechol and dopamine [27,46,47], which is attributed to Ca^2+^-mediated deprotonation of the catechol group and subsequent 1e^–^ oxidation by O_2_ [46]. Ca^2+^ ions also associate with and stabilize semiquinone radicals, as has been observed for various multivalent cations in eumelanin [48]. The strong increase of Δ*A*_337nm_ with [CaCl_2_] supports this proposed catechol-cation interaction in PDA films (Figure 2a and Figure S3). The proportional increase in *ΔA*_337nm_ between pH = 7.5 and 9.5 (Figure 2b and Figure S4) also indicates an underlying mechanism dependent on catechol deprotonation (p*K*_a_~9) [49]. Interestingly, the radical population in synthetic DL-DOPA melanin (as measured by electron paramagnetic resonance) exhibits a qualitatively equivalent dependence on pH as that shown in Figure 2b, further supporting the assignment of Δ*A*_337nm_ to semiquinone radicals [50]. Δ*A*_487nm_ exhibits an equivalent dependency with pH and [CaCl_2_] as Δ*A*_337nm_ (Figure S5) except for a difference at pH = 2 which we cautiously speculate may be related to protonation of preexisting semiquinones [51] and its effect on intermolecular stacking in the film [52].

Aqueous solutions of redox-inactive cations can be used as post-processing buffers to control the composition of nascent PDA films. Alkaline NaCl solutions accelerate oxidation of pre-deposited PDA films compared to Tris buffer alone (Figure S6). The spectra recorded from films incubated in NaCl solutions exhibit suppressed absorbance values and less-defined peaks compared to PDA films incubated in CaCl_2_, which is likely due to relatively weak interaction of monovalent cations with catechols compared to divalent cations. Films incubated in alkaline MgCl_2_ solutions, in contrast to NaCl, do produce a spectrum of similar intensity to CaCl_2_ (Figure S6b). There are two weak convoluted peaks at ca. *λ*_Abs_ = 458 and 310 nm which suggests a slightly different distribution of oxidation products or cation-catecholate interaction compared to films in CaCl_2_ solutions. Overall, the accelerated spectral evolution of PDA films in the presence of NaCl, MgCl_2_, and CaCl_2_ solutions show that redox-inactive cations modify the constituent molecules in PDA films.

**Figure 3:**
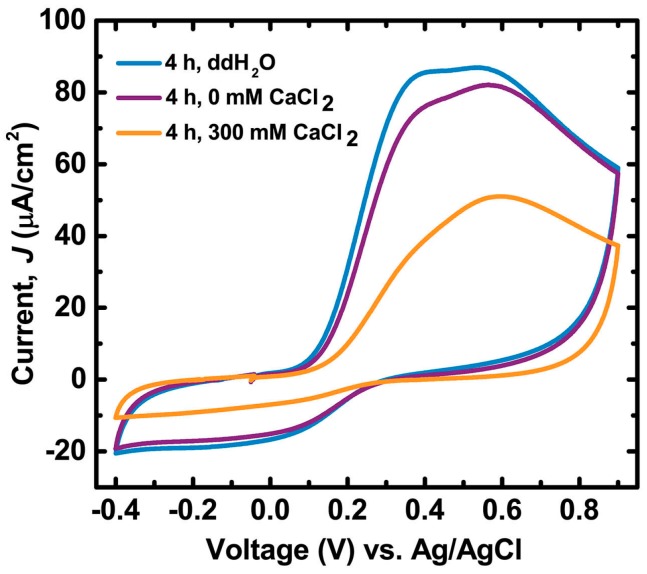
First cycle voltammograms of PDA films on indium tin oxide (ITO) that were incubated for 4 h in either ddH_2_O, 0 mM CaCl_2_ 50 mM Tris buffer pH = 9.5, or 300 mM CaCl_2_ 50 mM Tris buffer pH = 9.5. Electrolyte consisted of 180 mM sodium diphosphate–citric acid buffer and 100 mM NaCl at pH = 7.0 purged with N_2_. Scan rate was 30 mV/s. See Figure S8 for cycles > 1.

Cation-mediated oxidation of PDA films is supported by cyclic voltammetry (Figure 3). The midpoint between the onset of the oxidation and reduction curves in the voltammogram is approximately 180 mV (vs. Ag/AgCl), consistent with a dopamine/quinone redox couple at pH = 7.0 [40]. The films incubated in 300 mM CaCl_2_ solutions (pH = 9.5) for 4 h exhibited a total oxidative charge transfer only 56% of that of films incubated in equivalent pH without CaCl_2_ (4.9 ± 0.2 mC vs. 8.7 ± 0.4 mC, respectively). The decrease in oxidation capacity in CaCl_2_-incubated films is likely not due to mass loss (i.e., film dissolution) since there was no obvious morphological damage to the films and the film thickness did not decrease during incubation (Figure S7 and Table S1). Therefore, the relative decrease in oxidative charge transfer in PDA films incubated in CaCl_2_ solutions compared to those incubated without CaCl_2_ is evidence that Ca^2+^ ions promote oxidation of PDA.

### 2.2. Alkaline Calcium Solutions Decrease Dopamine Content in PDA and Generate Carboxylic Acid Groups

The altered molecular composition of PDA exposed to redox-inactive cations has important implications for PDA’s function as a metal sorption material [10,11,12,13]. The 2e^−^ oxidation of DHI produces 5,6-indolequinone which can tautomerize to quinone-imine [53,54]. In addition, H_2_O_2_ in alkaline solutions—generated during dopamine oxidation—can transform DHI species into pyrrole scaffolds with pendant carboxylates [22]. Quinone-imine and carboxylates may compete with catechols for metal cations and increase binding capacity in PDA since p*K*_a,quinone-imine, carboxylate_ < p*K*_a,dopamine_ [53,55]. These species also contribute negative stationary charges in PDA films that can affect ion transport within the film [56,57].

**Figure 4:**
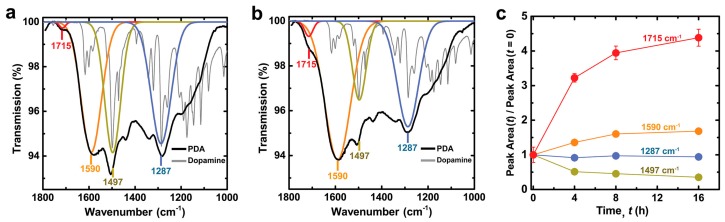
Attenuated total reflectance-infrared (ATR-IR) spectra of PDA films that were incubated in 300 mM CaCl_2_ 50 mM Tris buffer pH = 9.5 for: (**a**) 0 h; and (**b**) 4 h. The spectrum of stock dopamine powder is also shown and scaled for clarity; (**c**) Change in ATR-IR peak area vs. incubation time relative to the value for the pristine PDA film. See Figures S9 and S10 for data at 8 and 16 h and additional deconvoluted peaks. Data are represented as the average ± SD (*n* = 6 measurements at random locations on the sample).

The Ca^2+^-induced oxidation of PDA films decreases dopamine content and generates carboxylic acid groups, as supported by deconvoluted ATR–IR spectra (Figure 4). The large deconvoluted band at 1590 cm^−1^ for PDA is assigned to aromatic ring vibrations of various species in the film. The clear overlap of a deconvoluted band at 1497 cm^−1^ in PDA with the strong 1499 cm^−1^ aromatic ring vibration of dopamine [58] suggests that this band indicates uncyclized dopamine content in PDA. Incubating PDA in alkaline CaCl_2_ solution lowers the intensity at 1497 cm^−1^ with a concomitant increase in intensity at 1590 cm^−1^, and this is attributed to oxidation and cyclization of dopamine into heterocyclic products. Interestingly, the relative intensity of the 1497 cm^−1^ band after 4 h compared to pristine PDA (51% ± 2%) is equivalent to the relative decrease in oxidative charge transfer observed after 4 h (53% ± 2%), supporting the assignment of the 1497 cm^−1^ band to dopamine. The comparatively small decrease in the intensity of the 1287 cm^−1^ band, assigned to C–O stretching, is attributed to an insignificant effect of dopamine cyclization on the C–O stretching peak position within the deconvoluted band. A small band at 1715 cm^−1^ increases with increasing incubation time, which in light of zeta potential measurements is attributed to generation of pyrrole carboxylic acids (discussed below). The aforementioned changes in features observed in IR spectra with increasing time in alkaline CaCl_2_ solution are comparable to those observed in PDA created from dopamine with increasing concentrations of chemical oxidant [19]. This congruence further supports the interpretation that Ca^2+^ cations promote in situ oxidation of PDA films.

**Figure 5:**
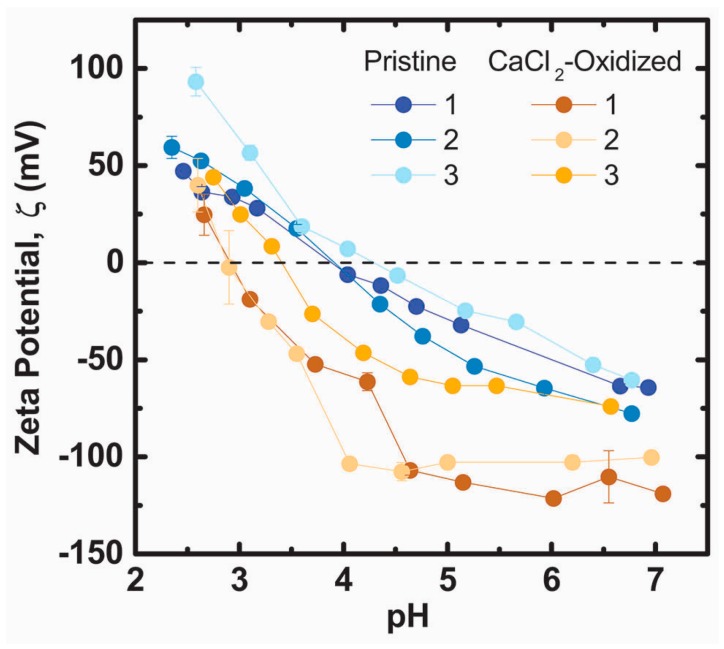
ζ potential titrations of pristine PDA films and PDA films that were incubated in 300 mM CaCl_2_ 50 mM Tris buffer pH = 9.5 for four hours. Numbers 1–3 designate unique samples.

The formation of pyrrole carboxylic acid groups as a downstream oxidation product in CaCl_2_-oxidized PDA films is supported by zeta potential measurements. The zeta potential of pristine PDA is negative above pH = 4 and becomes less negative as the pH is decreased (Figure 5). Catechol and amine groups in the PDA films are fully protonated at pH = 7 [49]. Therefore, the loss of negative surface charge below pH = 7.0 in pristine PDA is attributed to protonation of a variety of other species such as quinone imine (p*K*_a_: 6.3) [53], or pyrrole carboxylic acids. The primary amines on uncyclized dopamine incorporated in PDA films also contribute to the net positive *ζ* at pH < 4. The average isoelectric point of pH = 4.0 ± 0.2 for the three pristine PDA samples agrees with a previous report [59]. The value of *ζ* becomes more negative as PDA films are incubated in 300 mM CaCl_2_ solutions resulting in a lowered average isoelectric point of 3.1 ± 0.3. A new inflection point appears around pH = 4.5 below which *ζ* becomes less negative. The pH value of this inflection point suggests protonation of carboxylic acid moieties, while the absence of an inflection point at pH = 6.3 suggests that quinone-imine species do not contribute significantly to *ζ* for CaCl_2_-oxidized PDA films [59]. Protonation of residual dopamine-semiquinone anions (p*K*_a_: 4.7) [60] generated in CaCl_2_-oxidized films (Figure 1a) may also contribute to the new inflection point. The more negative surface charge in oxidized PDA films is therefore attributed to pyrrole carboxylic acid groups, which are known downstream products of PDA oxidation [22], and dopamine-semiquinone.

### 2.3. Films Incubated in Alkaline CaCl_2_ Solutions Exhibit Enhanced Iron Chelation

The altered molecular composition of CaCl_2_-oxidized PDA films increases iron chelation as measured by Raman spectroscopy. Raman spectroscopy can identify iron-catechol chelates in *Sepia* melanin [61] and mussel byssal threads [62] which contain equivalent structural components (dihydroxyindoles and catecholamines) to PDA. The spectrum of PDA (Figure S11) is dominated by two broad convoluted peaks at ca. 1360 and 1570 cm^−1^ which are characteristic of disordered aromatic carbon materials [63]. Deconvolution of the region between 1050–1800 cm^−1^ revealed 6 component vibrational bands similar to that of eumelanin (Figures S12 and S13) [64,65]. The two most prominent bands centered at 1587 and 1356 cm^−1^ are assigned to the in-plane parallel displacement of sp^2^-bonded carbons (“G band”) and the breathing vibration of 6-carbon aromatic rings (“D band”), respectively [63]. The overall spectra and individual components of the deconvoluted spectra are observed in various disordered aromatic carbon materials such as humic acids and carbonized biopolymers [66,67,68,69]. A low intensity band centered at 460 cm^−1^, which is attributed to torsion of –OH groups in catechols, is also present in the Raman spectrum of PDA [61,70].

**Figure 6:**
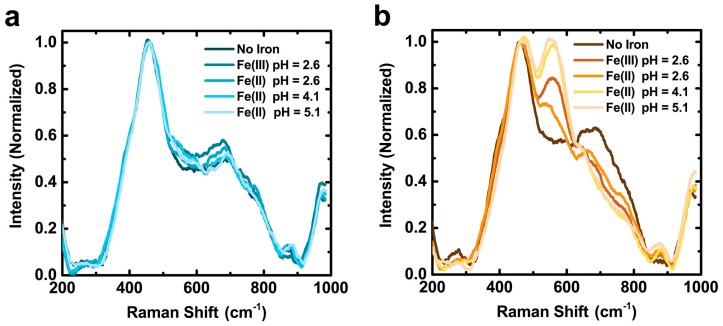
Raman spectra of PDA films before and after incubation in 5 mM ferric or ferrous chloride solutions for 100 min, labeled as Fe(III) or Fe(II), respectively: (**a**) Pristine PDA films; and (**b**) PDA films that were incubated in in 300 mM CaCl_2_ 50 mM Tris buffer pH = 9.5 for 4 h. The band centered at 460 cm^−1^ corresponds to –OH torsion. The band at 555 cm^−1^ visible in (**b**) is due to vibration of the five-membered chelate ring of iron with catechol. See Figures S11–S15 for full Raman spectra between 200 and 2000 cm^−1^ and quantification of band intensities.

CaCl_2_-oxidized PDA films exhibit clear enhanced iron chelation after incubation in iron solutions. The Raman spectra of pristine films remain unchanged before and after incubation in all iron solutions. Conversely, CaCl_2_-oxidized PDA films generate two features: a new band at 555 cm^−1^; an increase in intensity at 1482 cm^−1^ (Figure 6 and Figure S11). These signatures observed in PDA are also detected in *Sepia* melanin [61]. When *Sepia* melanin is enriched with Fe^3+^ a new band appears at 570 cm^−1^ with similar intensity to the 460 cm^−1^ band, and the intensity increases in the convoluted 1470 cm^−1^ region. The two intensified bands imply that CaCl_2_-oxidized PDA films exhibit increased iron chelation capacity compared to pristine counterparts. The chelate vibrations of Fe-enediolate complexes are observed in the 500–600 cm^−1^ region in a variety of systems [62,71,72,73]. The 555 cm^−1^ band is assigned to the 5-membered chelate ring of catechol moieties bound to iron in PDA. Additionally, a band between 1480–1490 cm^−1^ is also indicative of catecholic chelates with iron in solution or nanoparticle surfaces [73,74,75,76]. The intensity of the 1482 cm^−1^ band follows the same trend with pH and film condition as the 555 cm^–1^ band (Figures S14 and S15), indicating that both bands serve as indicators of increased iron chelation capacity in CaCl_2_-oxidized films compared to pristine PDA films.

Elevated iron chelation capacity in CaCl_2_-oxidized films is attributed to several factors. Previous electrochemical studies on PDA films have revealed that at pH < 4, PDA films are impermeable to multivalent cations due to a net positive charge in the film [56,57]. Pristine films exhibit more positive zeta potentials compared to CaCl_2_-oxidized films which could account for the lack of detectable iron chelation in pristine films. However, altered surface charges cannot fully account for the differences in iron chelation as iron chelation is observed in CaCl_2_-oxidized films at pH = 2.6 where *ζ* > 0, and not observed in pristine PDA films at pH = 5.1 where *ζ* < 0. The most significant factor affecting iron chelation is likely the difference in iron-binding affinity between dopamine and DHI units. DHI has a higher binding affinity for Fe^3+^ between pH 3–10 compared to free dopamine [77]. Additionally, bis-coordinated catechol-Fe^3+^ species are stable over a wider pH range for DHI compared to dopamine. Alkaline solutions of CaCl_2_ bias the products of the PDA synthetic pathway towards downstream oxidation products including DHI units within PDA oligomers. Therefore, the increase in catechol-Fe^3+^ chelation is primarily attributed to increased concentrations of DHI units in post-processed PDA films.

The generation of downstream oxidation products of dopamine (e.g., DHI and pyrrole carboxylic acids) suggests that cation sorption functionality can be controlled by post-processing. Ion exchange studies on *Sepia* melanin revealed that Mg^2+^ and Ca^2+^ cations have a greater passive affinity for carboxylic acid groups in melanin compared to catechol groups, and the converse for Fe^3+^ [55]. Therefore, CaCl_2_-induced oxidation of PDA may increase PDA’s passive affinity for group II/I cations via pyrrole-2,3,dicarboxylic acid (PDCA) units while simultaneously enhancing chelation of Fe^3+^ via DHI units or potentially PDCA units. This is in contrast to a more actively-controlled (i.e., electrically biased) affinity for group II cations in pristine PDA films [14]. Post-processing of PDA films using controlled buffer solutions has advantages over electrochemical control of cation chelation because this strategy is applicable to a wider range of substrates including insulating materials. Additionally, cation transport through PDA films can be enhanced by more carboxylic acid groups, as discussed previously in the context of more negative ζ potential of the films [56,57]. Therefore, post-processing of PDA films can potentially be used to modulate the metal sorption properties of PDA films for application specific coatings. Prospective applications include novel ion-exchange membranes or separation layers for electrochemical storage systems [12,13,14,78]. Ambient application conditions may also modulate device properties over time. The typical Ca^2+^ concentrations (0.4–5 mM) and pH (6.4–9.2) of rivers and lakes [79] could accelerate the oxidative evolution of PDA ion-exchange coatings in a manner equivalent to the films studied herein, albeit over longer time scales (Figure 2 and Figures S3–S5).

In addition to selective control over cation binding, post-processing techniques can also control the amount of uncyclized dopamine in PDA films. Higher surface densities of primary amines may be useful for bioconjugation techniques that are orthogonal to catechols and carboxylates [10,80,81]. Non-cyclized primary amines may also afford strategies to control the mechanical properties of PDA films through chemical crosslinking. Taken together, the post-processing capabilities afforded by multivalent cation buffers can potentially fine-tune the properties of PDA films depending upon the application of interest. The ambient salt and mildly basic pH of body fluid ([Ca^2+^] = 1.3 mM, [Mg^2+^] = 1.0 mM, and [Na^+^] = 142 mM, pH = 7.4) [82] may also affect the properties of PDA-incorporated biomedical devices over long time periods (days/weeks).

## 3. Materials and Methods

### 3.1. Materials

Dopamine hydrochloride (98%), iron(III) chloride hexahydrate (>99%), iron(II) chloride tetrahydrate (>99%) sodium chloride (>99%) and citric acid (>99.5%) were purchased from Sigma-Aldrich (St. Louis, MO, USA) and used as received. Sodium bicarbonate (>99%), sodium carbonate (>99%), tris(hydroxymethyl) aminomethane (Tris), sodium phosphate dibasic (99.2%) and magnesium chloride hexahydrate (>99%) were purchased from Fisher Scientific (Hampton, NH, USA) and used as received. Calcium chloride dihydrate (>99%) was purchased from BDH VWR International (Radnor, PA, USA). Water was purified (18.2 MΩ·cm) using Direct-Q 3 UV-R system (EMD Millipore, Billerica, MA, USA). Silicon wafers with native oxide were purchased from Silicon Quest International (San Jose, CA, USA; 1” diameter, phosphorus doped). Indium tin oxide (ITO) pieces were purchased from University Wafer (Boston, MA, USA; 20 Ω/sq; ITO on glass).

### 3.2. PDA Film Preparation and Iron Binding

Silicon and ITO substrates were cleaned by sonication in acetone, followed by isopropyl alcohol and then rinsed with de-ionized water (ddH_2_O). Substrates were then cleaned by UV-ozone (30 mW/cm^2^, 5 min; Jelight, Irvine, CA, USA). PDA films were prepared by dissolving 2 mg/mL dopamine hydrochloride in 200 mL of 50 mM bicarbonate buffer at pH = 8.5. Pre-cleaned substrates were incubated in dopamine solutions in ambient air and rotational agitation (65 rpm). After 24 h the substrates were rinsed and incubated in a refreshed deposition solution for another 24 h and a total 48 h deposition time. The substrates were then rinsed and placed in ddH_2_O for 24 h then dried under a stream of N_2_.

PDA films were incubated in 3.2 mM HCl (pH = 2.5) for 15 min after exposure to post-processing solutions (CaCl_2_ + Tris, Tris, and ddH_2_O only) and then washed with ddH_2_O to remove precipitates and equilibrate the pH within the films. The pH values of all sample solutions were measured using an Ag/AgCl electrode pH probe (Hach, model 5014T; Loveland, CO, USA).

For iron chelation assays, PDA films were incubated in either ddH_2_O or 300 mM CaCl_2_ (pH = 9.5) for 4 h, then in pH = 2.5 HCl for 15 min, and finally 5 mM FeCl_2_ or FeCl_3_ solutions for 100 min in ambient atmosphere. FeCl_2_ was used to investigate iron chelation at pH > 4 where Fe^3+^ ions precipitate as hydroxide species [83]. The Fe^2+^ oxidation state is predominant within the 100 min time scale for the FeCl_2_ solutions in ambient oxidizing conditions [84,85].

### 3.3. Spectroscopic and Electrochemical Characterization of PDA Films

Ultraviolet-Visible (UV-Vis) spectra of hydrated PDA films on 0.9 × 2.5 cm^2^ ITO substrates were recorded (UV-2600, Shimadzu; Kyoto, Japan) in various buffered salt solutions with a pristine ITO substrate and equivalent buffered salt solution in the reference cell. After incubating the PDA films for 3 min in the desired solution (to allow for solution permeation and pH equilibration), the spectra of the films were recorded every 83 s for several hours.

Fourier transform infrared (FTIR) spectra of the PDA films were recorded via attenuated total reflectance (ATR) technique (Frontier, PerkinElmer; Waltham, MA, USA). Raman spectra (NTegra Spectra; Tempe, AZ, USA) were recorded with a 532 nm laser. Each measurement was done at unique single point of spot size ~1 µm diameter at 1 mW power for 50–60 s. Peak deconvolution was performed with OriginLab software (Northampton, MA, USA).

Zeta potentials of PDA films were calculated by measuring the streaming potential near a rotating disk [86] (PDA film on SiO_2_) at different pH with a custom-built ZetaSpin apparatus [87].

Cyclic voltammetry was performed in a conventional three-electrode setup with saturated Ag/AgCl reference electrodes (Koslow Scientific; Englewood, NJ, USA) and platinum mesh as counter electrode. Tests were performed in 180 mM citric acid-sodium phosphate buffer (pH = 7.0) with 100 mM NaCl supporting electrolyte that had been purged for 1 h N_2_. Scans were swept between −0.4 V and 0.9 V at 30 mV/s using an Interface 1000 potentiostat (Gamry Instruments; Warminster, PA, USA). Submerged PDA film area was 4.75 cm^2^ and current densities are reported based on this value.

### 3.4. Morphological Characterization of PDA Films

Film thicknesses and morphology were measured using atomic force microscopy (NT-MDT NTegra AFM; Tempe, AZ, USA) in tapping mode. Large range scans were 9 × 9 µm^2^ at 0.8 Hz (NT-MDT NTegra AFM; Tempe, AZ, USA; *k* = 25–95 N/m, tip radius = 35 nm). High resolution scans were recorded at areas of 0.9 × 0.9 µm^2^ at 2 Hz (Budget Sensors, Sofia, Bulgaria; *k* = 5 N/m, tip radius <1 nm). Film thickness was determined by scratching the films and measuring the height profile with atomic force microscopy (AFM) [17].

## 4. Conclusions

Post-processing of PDA films using redox-inactive metal cations can bias oxidative dopamine pathways to produce downstream products. (e.g., DHI and pyrrole carboxylic acids as opposed to dopamine). The enhanced iron chelating ability of the oxidized PDA films is attributed to a more negative ζ potential and greater binding affinity for Fe^3+^ in catechols found in DHI compared to dopamine. These results emphasize the practical considerations and limitations associated with the non-equilibrium state and heterogeneity of pristine PDA films. Technological translation of PDA films must account for the evolution of PDA’s chemical behavior over time in different environmental conditions. PDA’s heterogeneity also enables a broad range of control over its properties, and group I/II cation solutions can act as a post-processing strategy to tune the chemical composition of PDA films. The balance between upstream products such as uncyclized dopamine can be balanced with downstream products such as DHI. Therefore, while the chemical evolution of PDA films during application may not be completely avoidable, their properties upon device fabrication (e.g., surface charge, cation sorption, redox capacity, functional groups) can determine the initial operating behavior and functional lifespan of the device. This technique could improve reproducibility of specific PDA film properties and ultimately accelerate the use of PDA as a functional material.

## Supplementary Materials

Supplementary materials can be found at www.mdpi.com/1422-0067/18/1/14/s1.

## Abbreviations

PDA	Polydopamine
DHI	5,6-Dihydroxyindole
PDCA	Pyrrole-2,3,dicarboxylic acid
UV-Vis	Ultraviolet-visible
ATR-IR	Attenuated total reflectance-infrared
AFM	Atomic force microscopy
