# Supplementary material for: Calcium-Mediated Control of Polydopamine Film Oxidation and Iron Chelation
Source: Int J Mol Sci. 2016 Dec 22;18(1):14. doi: 10.3390/ijms18010014 (PMC5297649; doi:10.3390/ijms18010014)
Supplement: Supplementary file 1 [file ijms-18-00014-s001.pdf]

Supplementary Materials: Calcium-Mediated Control of Polydopamine Film Oxidation and Iron Chelation

Luke Klosterman and Christopher J. Bettinger

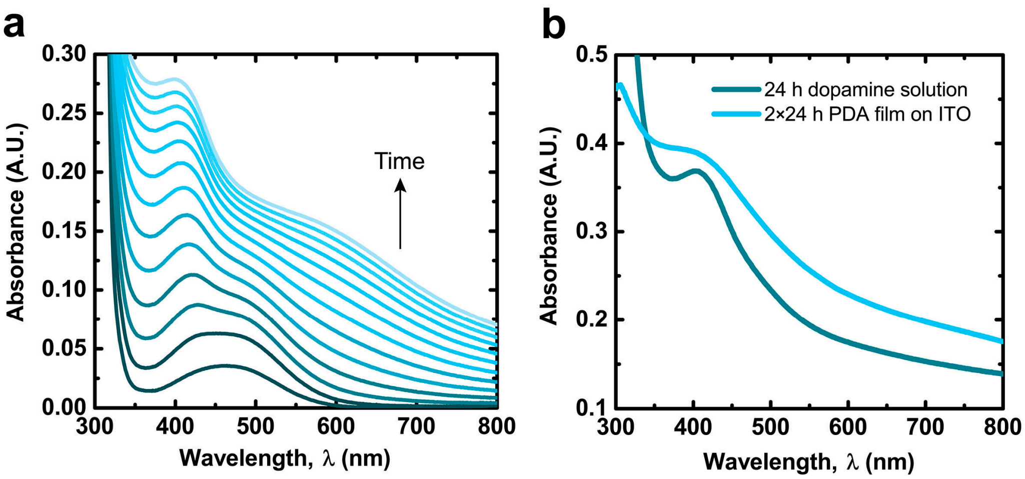

Figure S1. (**a**) Ultraviolet-visible (UV-Vis) spectra of 2 mg/mL dopamine solution in pH = 8.5 50 mM bicarbonate buffer. 30 s after solution preparation, scans were taken every 83 seconds for a total of ~18 min; (**b**) Spectra of dopamine solution after 24 h (diluted 1:10 vol) and polydopamine (PDA) film on indium tin oxide (ITO) that sat in the dopamine solution for 24 h then 24 h again in a refreshed solution.

The spectrum of pristine polydopamine (PDA) film shows a monotonic increase in absorption towards the ultraviolet region except for a peak at λ_{Abs} = 405 nm. This peak appears in the first several minutes of dopamine oxidation (Figure S1a). The peak's origin is not due to either dopaminochrome (λ_{Abs} = 475 nm) or dopamine-quinone (λ_{Abs} = 388 nm) [40]. Considering that the peak appears to evolve out of the initial dopaminochrome peak it may be due to dimers of dopaminochrome and 5,6,-dihydroxyindole (DHI) which absorb around λ_{Abs} = 400–450 nm [1].

The ultraviolet-visible (UV-Vis) spectrum of pristine PDA films is qualitatively similar to the deposition solution (Figure S1b), indicating that similar products are formed in the films and the solution.

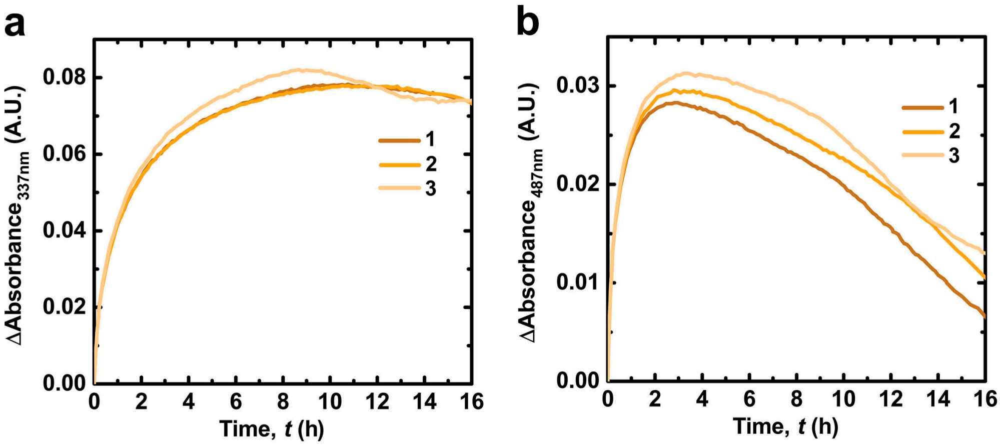

Figure S2. Absorbance vs. time for the peaks observed in subtracted PDA film spectra on three different samples. Incubation solution was 300 mM $CaCl_2$ 50 mM tris(hydroxymethyl) aminomethane (Tris) buffer pH = 9.5. (**a**) λ_{Abs} = 337 nm; (**b**) λ_{Abs} = 487 nm.

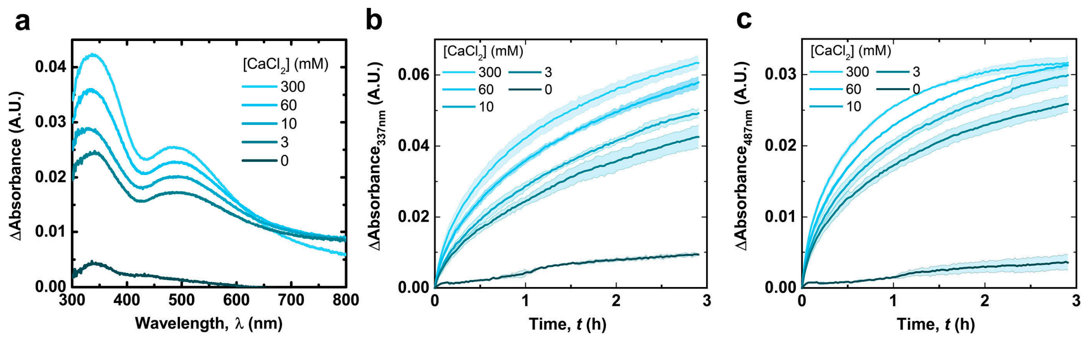

Figure S3. (**a**) Subtracted spectra of PDA films after 1 h in 50 mM Tris buffer pH = 9.5 in different concentrations of $CaCl_2$. Average of two representative samples; (**b**) Absorbance at λ_{Abs} = 337 nm over 3 h. The light shaded regions correspond to the range defined by average ± SD (n = 2); (**c**) Absorbance at λ_{Abs} = 487 nm.

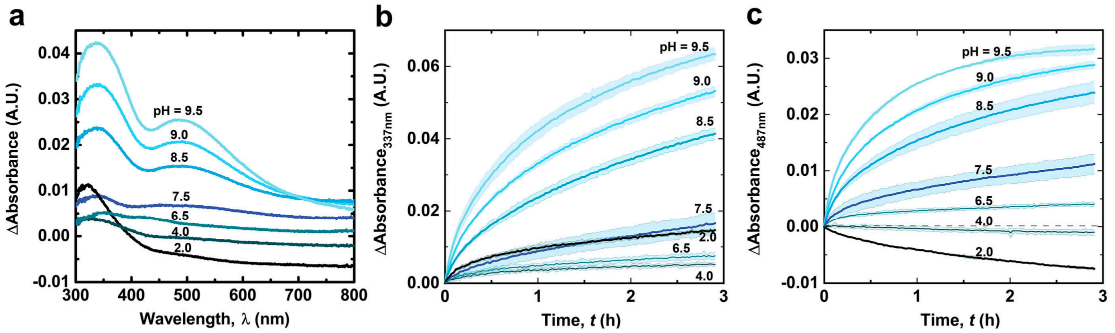

Figure S4. (**a**) Subtracted spectra of PDA films after 1 h in 300 mM [$CaCl_2$] 50 mM Tris buffer at different pH. Average of two representative samples; (**b**) Absorbance at λ_{Abs} = 337 nm over 3 h. The light shaded regions correspond to the range defined by average ± SD (n = 2); (**c**) Absorbance at λ_{Abs} = 487 nm over 3 h.

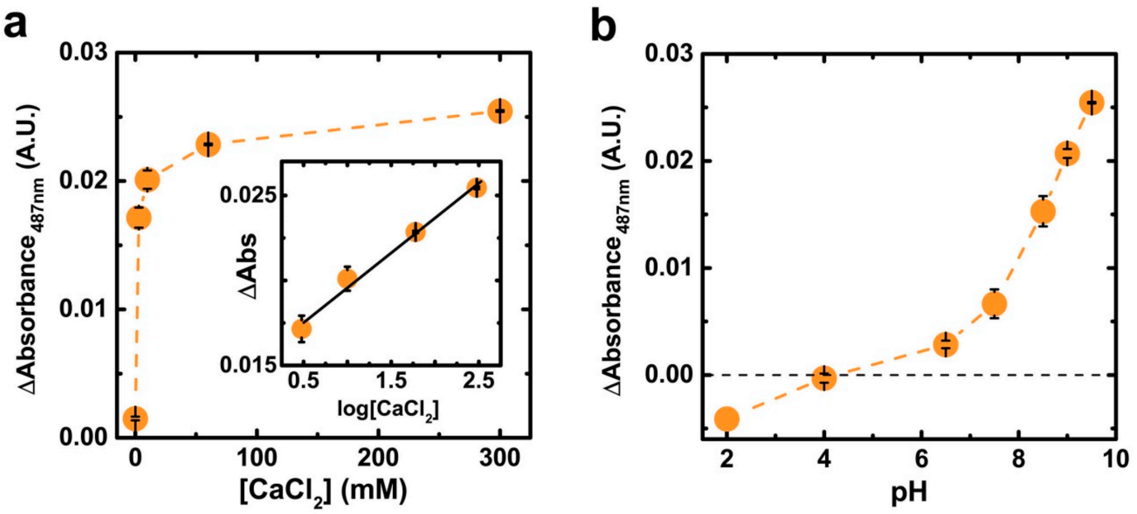

Figure S5. (**a**) Subtracted spectra absorbance of PDA films at λ_{Abs} = 487 nm after 1 h as a function of $CaCl_2$ in 50 mM Tris buffer pH = 9.5; (**b**) Subtracted spectra absorbance at λ_{Abs} = 487 nm after 1 h as a function of pH in 300 mM $CaCl_2$ 50 mM Tris buffer. See Figure 2 for equivalent data at λ_{Abs} = 337 nm. Data are represented as the average ± SD (n = 2).

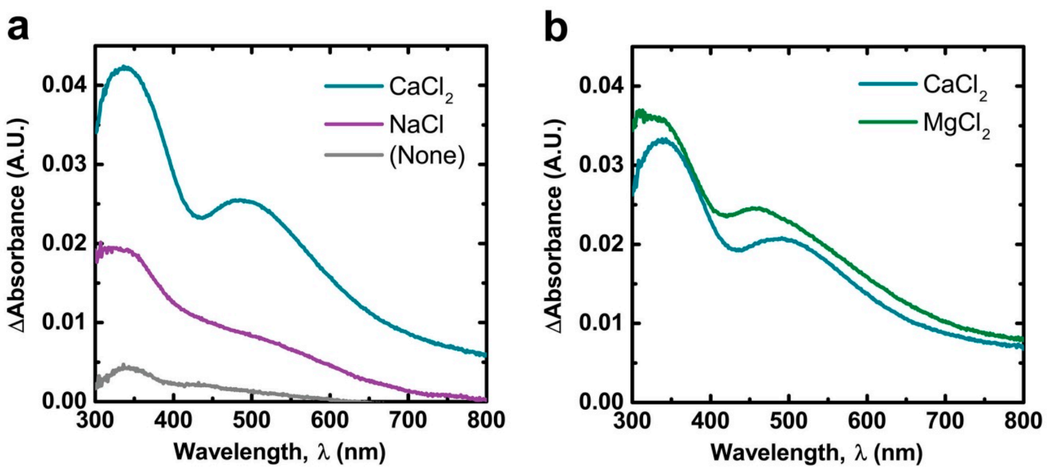

Figure S6. (**a**) Subtracted spectra of films after 1 h in 300 mM of indicated salt + 50 mM Tris buffer pH 9.5; (**b**) Subtracted spectra after 1 h in 300 mM of indicated salt + 50 mM Tris buffer pH 9.0. Spectra are averages of two samples.

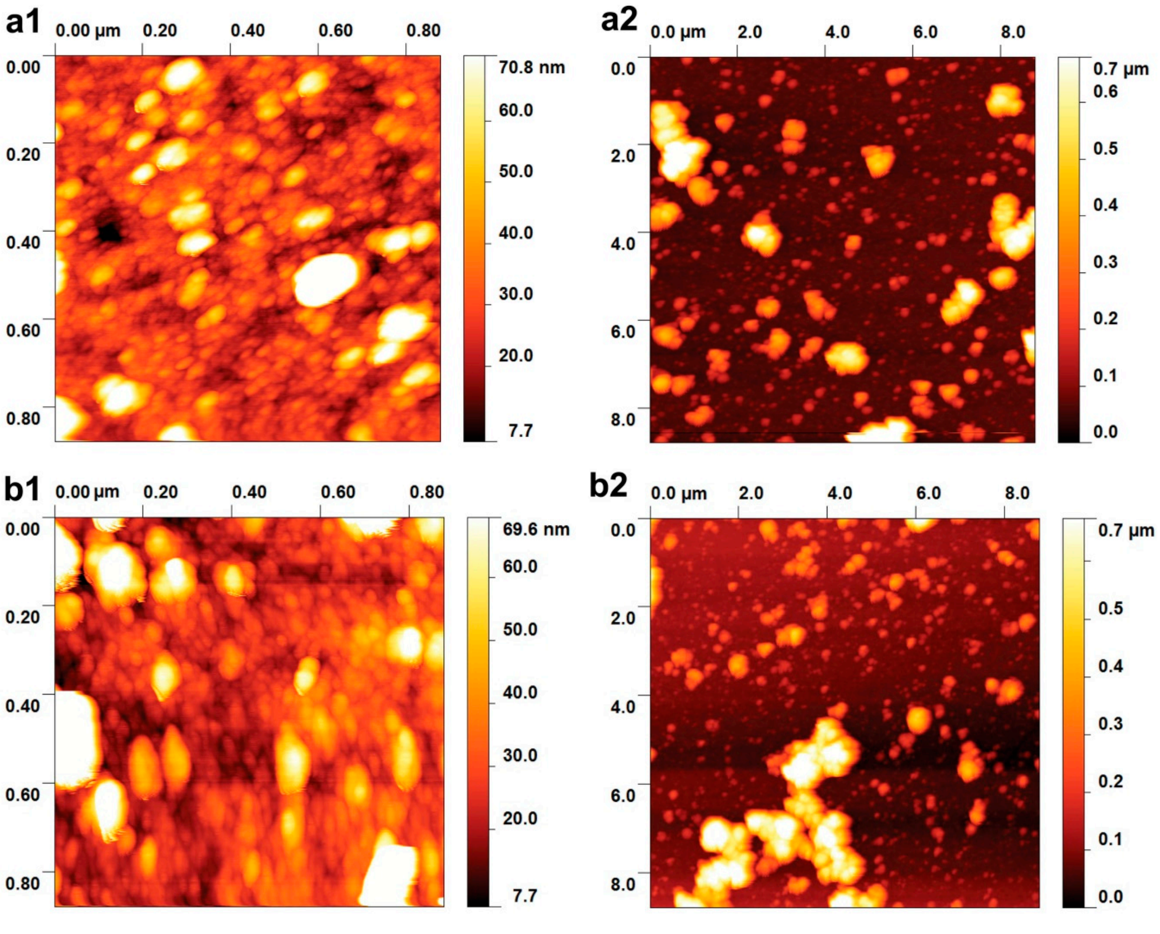

Figure S7. Atomic force microscopy (AFM) scans of (**a**) pristine film (**b**) film incubated in 300 mM [$CaCl_2$] 50 mM Tris buffer pH = 9.5 for 4 h.

The oxidation products of dopamine deposit on various substrates as conformal films shown in Figure S7. The films nucleate as 3-dimensional islands [17] which grow and coarsen until they impinge on each other forming a network of cohesive granules with a characteristic length scale of 20–50 nm in diameter. Micron-scale precipitates of PDA from the deposition solution also deposit on the surface of the granular film. The thicknesses of PDA films used in this study are shown in Table S1.

Table S1. Thickness of PDA films after different incubation times in 300 mM [$CaCl_2$] 50 mM Tris buffer solutions at pH = 9.5 Data are represented as the average ± SD (*n* = 6 measurements on each sample).

Incubation Time (h)	Thickness (nm)
0	59 ± 3
4	61 ± 6
8	60 ± 3
16	62 ± 6

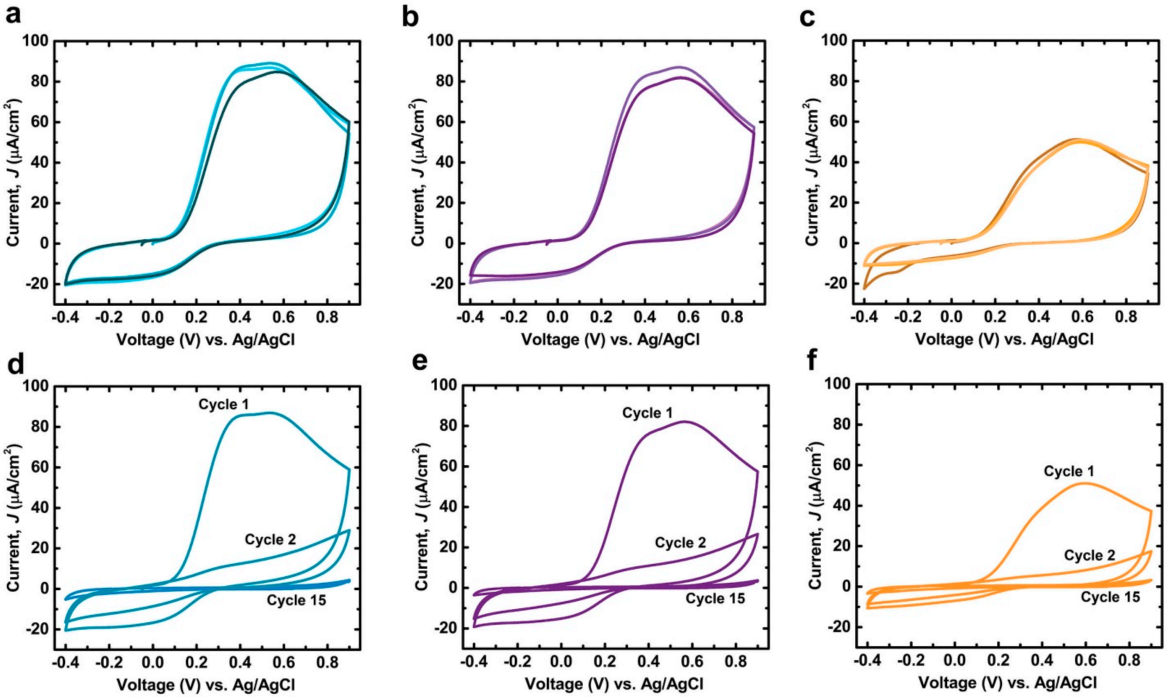

Figure S8. Cyclic voltammograms for three PDA film samples on ITO after they had been incubated for 4 h in (**a**) ddH_2O (**b**) 50 mM Tris buffer pH = 9.5; (**c**) 300 mM $CaCl_2$ 50 mM Tris buffer pH = 9.5. Also shown are cycles 2 and 15 for (**d**) ddH_2O (**e**) 50 mM Tris buffer pH = 9.5; (**f**) 300 mM $CaCl_2$ 50 mM Tris buffer pH = 9.5. Electrolyte consisted of 180 mM sodium diphosphate–citric acid buffer and 100 mM NaCl at pH = 7.0 purged with N_2. Scan rate was 30 mV/s.

The voltammograms of dopamine solutions at pH = 7.0 display the dopamine/quinone redox couple at $E^{\circ\prime}$ = 182 mV and the dopaminochrome couple at $E^{\circ\prime}$ = −228 mV [40]. The total oxidative current observed in PDA films up to 0.9 V (vs. Ag/AgCl) cannot be attributed to specific molecular species but may be due to the variable redox potentials of assorted oligomers in the films.

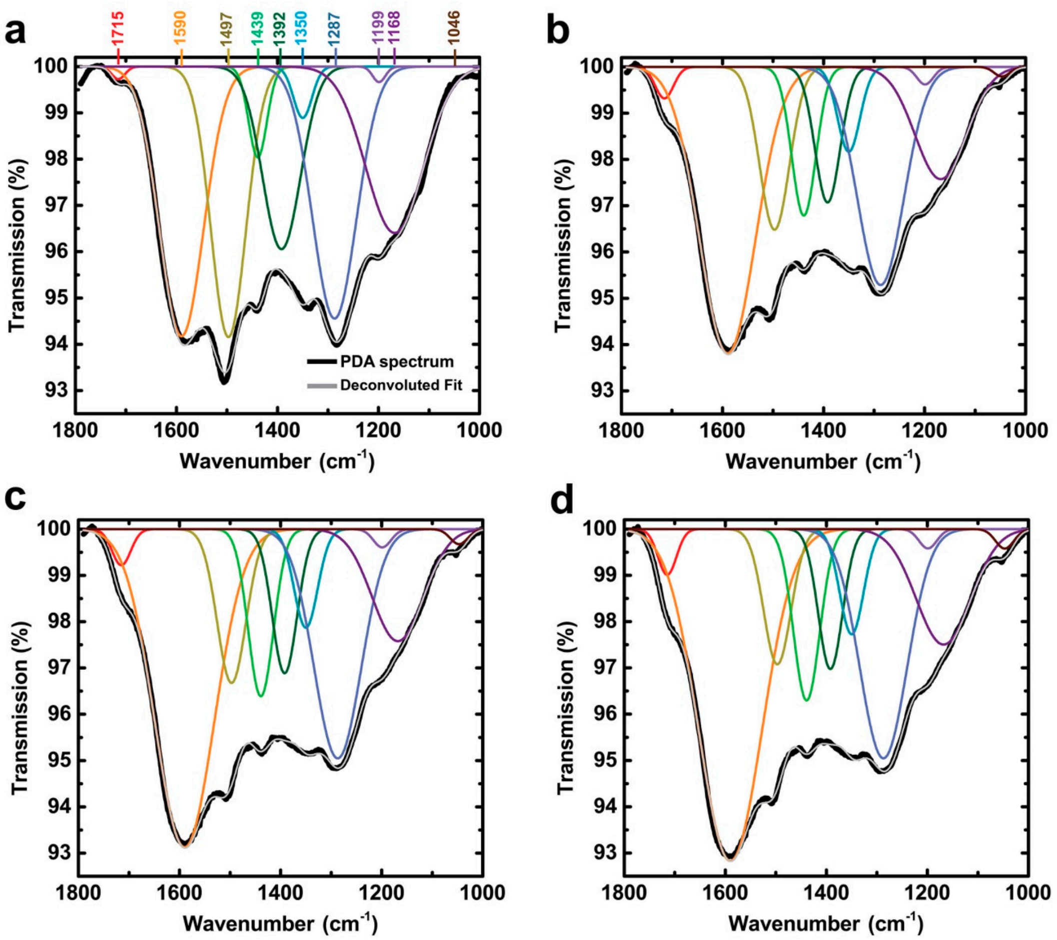

Figure S9. Attenuated total reflectance-infrared (ATR-IR) spectra and deconvolution of PDA films after different incubation times in 300 mM $CaCl_2$ 50 mM Tris buffer at pH = 9.5 (**a**) 0 h (**b**) 4 h (**c**) 8 h (**d**) 16 h. Spectra were corrected with a linear baseline between 1780 cm^{-1} and 1000 cm^{-1} (see spectra of PDA in reference [2] for comparison). Deconvolution was calculated with Gaussian peaks and peak positions were held constant between different deconvolutions.

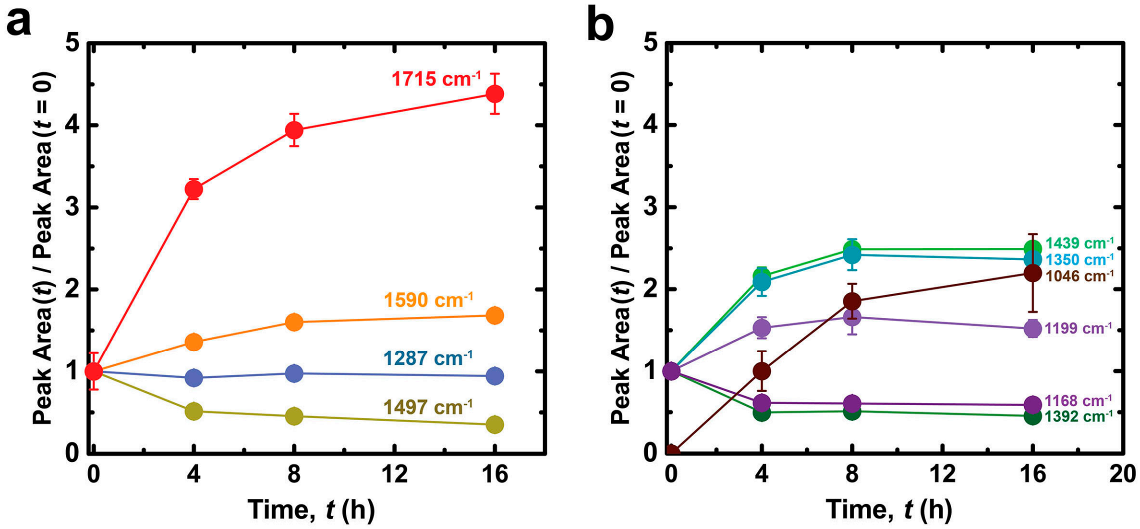

Figure S10. Change in ATR-IR deconvoluted peak area as shown in Figure S9 vs. incubation time relative to the value for the pristine PDA film. (**a**) Primary peaks of interest (also shown in Figure 4c); (**b**) Other peaks which remain unassigned. Data are represented as the average ± SD (n = 6 measurements at random locations on the sample).

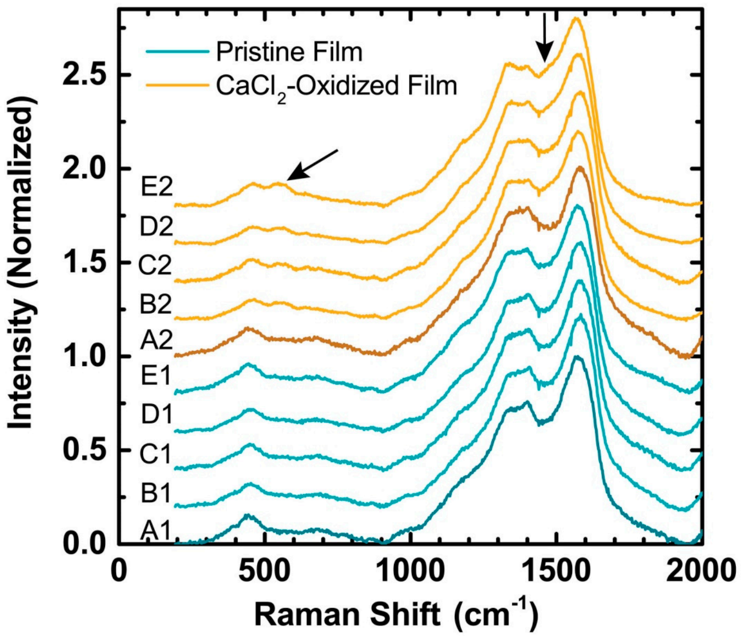

Figure S11. Raman spectra of PDA films before (**1**) and after (**2**) incubating in 5 mM iron solutions for 100 min. Arrows indicate changing features in spectra. (**A**) Films with no iron exposure; (**B**) $FeCl_3$ pH = 2.6 (**C**) $FeCl_2$ pH = 2.6 (**D**) $FeCl_2$ pH = 4.1 (**E**) $FeCl_2$ pH = 5.1.

The chelate vibrations of Fe-enediolate complexes are observed in the 500–600 cm^{-1} region in a variety of systems: 528 cm^{-1} for Fe[oxalate$_3$]$^{3-}$ and human tyrosine hydroxylase [71,72], 533 cm^{-1} for ferric tris-catecholate [73], 565 cm^{-1} for ferric-enterobactin [73], and 550 cm^{-1} for Fe-DOPA complexes [62].

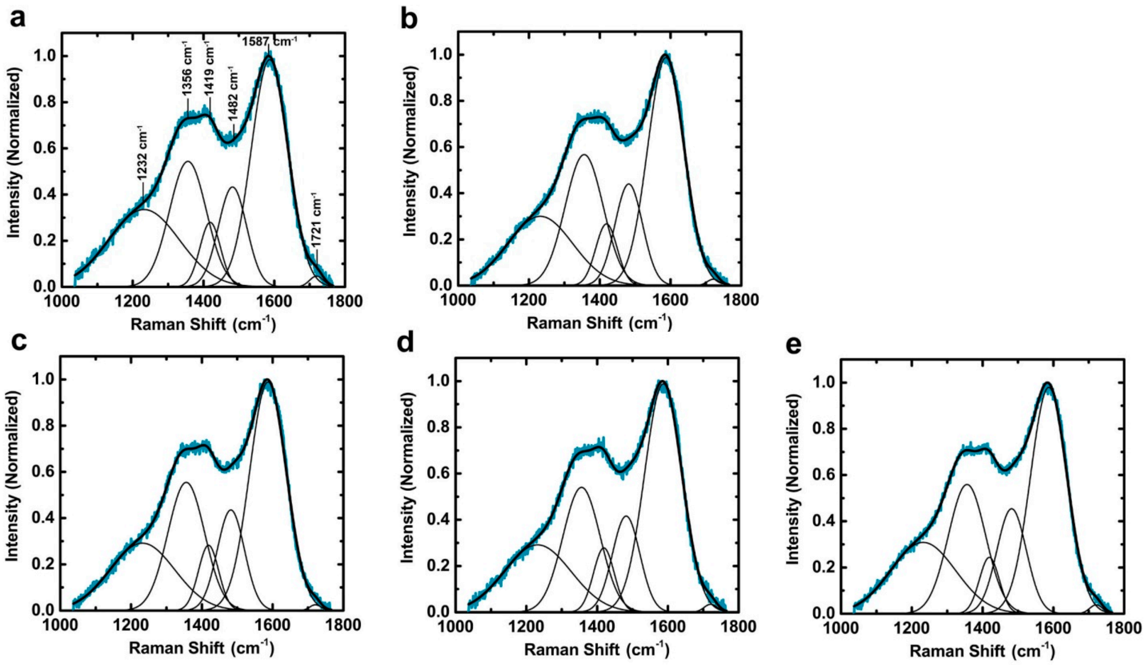

Figure S12. Deconvoluted Raman spectra of pristine PDA films before and after incubating in 5 mM iron solutions for 100 min. Peak positions were fixed at the same position for each deconvolution. (**a**) Films with no iron exposure; (**b**) $FeCl_3$ pH = 2.6; (**c**) $FeCl_2$ pH = 2.6; (**d**) $FeCl_2$ pH = 4.1; (**e**) $FeCl_2$ pH = 5.1.

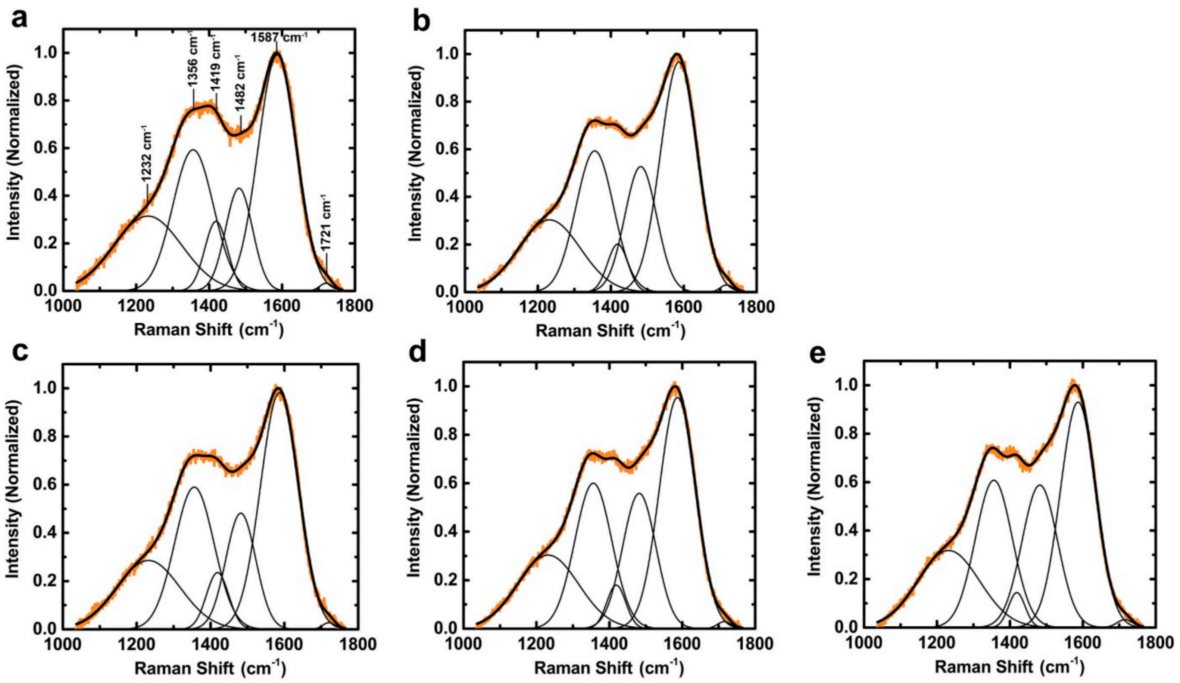

Figure S13. Deconvoluted Raman spectra of $CaCl_2$-oxidized PDA films before and after incubating in 5 mM iron solutions for 100 min. Peak positions were fixed at the same position for each deconvolution. (**a**) Films with no iron exposure; (**b**) $FeCl_3$ pH = 2.6; (**c**) $FeCl_2$ pH = 2.6; (**d**) $FeCl_2$ pH = 4.1; (**e**) $FeCl_2$ pH = 5.1.

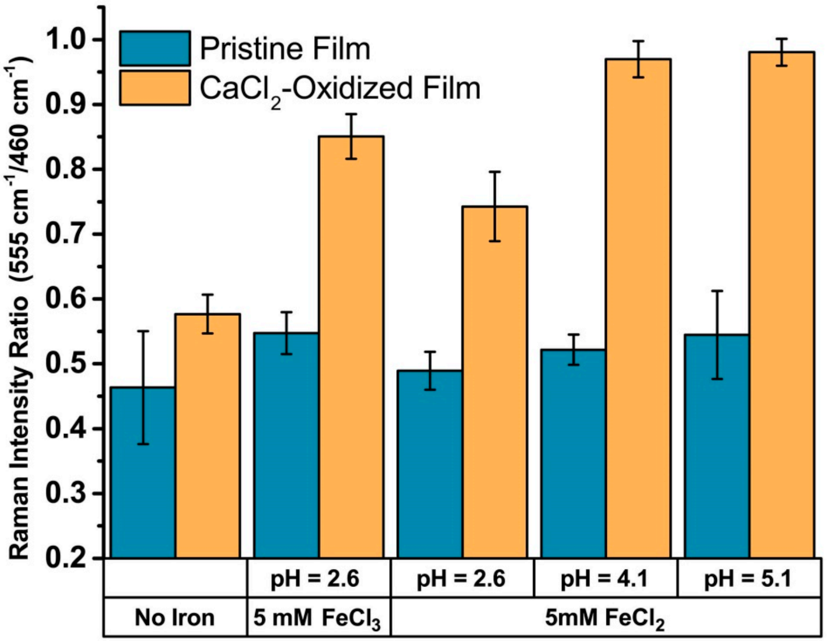

Figure S14. Ratio of the Raman intensity at 555 to 460 cm^{-1} for PDA films before and after incubating in 5 mM iron solutions for 100 min. Data are represented as the average ± SD (n = 5 measurements at random locations on the sample).

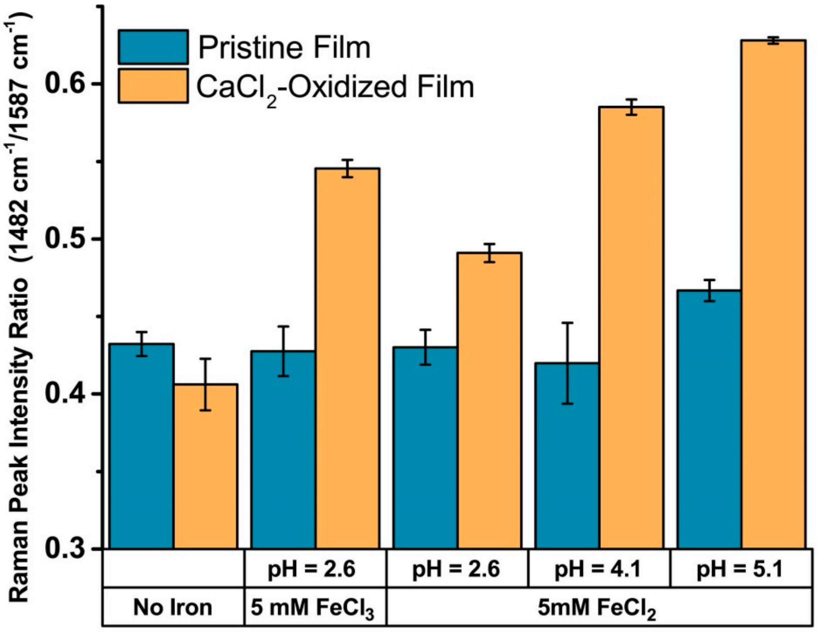

Figure S15. Ratio of the intensity of the 1482 cm^{-1} peak to the 1587 cm^{-1} peak for PDA films before and after incubating in 5 mM iron solutions for 100 min. Data are represented as the average ± SD (n = 5 measurements at random locations on the sample).

References

1. Pezzella, A.; Crescenzi, O.; Panzella, L.; Napolitano, A.; Land, E. J.; Barone, V.; D'Ischia, M. Free Radical Coupling of O-Semiquinones Uncovered. *J. Am. Chem. Soc.* **2013**, *135*, 12142–12149.
2. Luo, H.; Gu, C.; Zheng, W.; Dai, F.; Wang, X.; Zheng, Z. Facile Synthesis of Novel Size-Controlled Antibacterial Hybrid Spheres Using Silver Nanoparticles Loaded with Poly-Dopamine Spheres. *RSC Adv.* **2015**, *5*, 13470–13477.
